# Interaction of Ochratoxin A and Its Thermal Degradation Product 2′*R*-Ochratoxin A with Human Serum Albumin

**DOI:** 10.3390/toxins10070256

**Published:** 2018-06-22

**Authors:** Franziska Sueck, Miklós Poór, Zelma Faisal, Christoph G. W. Gertzen, Benedikt Cramer, Beáta Lemli, Sándor Kunsági-Máté, Holger Gohlke, Hans-Ulrich Humpf

**Affiliations:** 1 Institute of Food Chemistry, Westfälische Wilhelms-Universität Münster, Corrensstr. 45, 48149 Münster, Germany; f_suec01@uni-muenster.de (F.S.); cramerb@wwu.de (B.C.); 2 Department of Pharmacology, Faculty of Pharmacy, University of Pécs, Szigeti út 12, H-7624 Pécs, Hungary; poor.miklos@pte.hu (M.P.); faisal.zelma@gytk.pte.hu (Z.F.); 3 János Szentágothai Research Center, Ifjúság Útja 20, H-7624 Pécs, Hungary; lemli.beata@gytk.pte.hu (B.L.); kunsagi-mate.sandor@gytk.pte.hu (S.K.-M.); 4 Institute of Pharmaceutical and Medicinal Chemistry, Department of Mathematics and Natural Sciences, Heinrich Heine University Düsseldorf, Universitätsstr. 1, 40225 Düsseldorf, Germany; christoph.gertzen@hhu.de (C.G.W.G.); gohlke@uni-duesseldorf.de (H.G.); 5 John von Neumann Institute for Computing (NIC), Jülich Supercomputing Centre (JSC) & Institute for Complex Systems-Structural Biochemistry (ICS 6), Forschungszentrum Jülich GmbH, Wilhelm-Johnen-Str., 52425 Jülich, Germany; 6 Department of General and Physical Chemistry, University of Pécs, Ifjúság útja 6, H-7624 Pécs, Hungary; 7 Department of Pharmaceutical Chemistry, Faculty of Pharmacy, University of Pécs, Rókus utca 2, H-7624 Pécs, Hungary

**Keywords:** ochratoxin A, 2′*R*-ochratoxin A, mycotoxin, human serum albumin, dialysis, fluorescence spectroscopy, circular dichroism, high performance affinity chromatography, molecular modelling

## Abstract

Ochratoxin A (OTA) is a toxic secondary metabolite produced by several fungal species of the genus *Penicillium* and *Aspergillus*. 2′*R*-Ochratoxin A (2′*R*-OTA) is a thermal isomerization product of OTA formed during food processing at high temperatures. Both compounds are detectable in human blood in concentrations between 0.02 and 0.41 µg/L with 2′*R*-OTA being only detectable in the blood of coffee drinkers. Humans have approximately a fifty-fold higher exposure through food consumption to OTA than to 2′*R*-OTA. In human blood, however, the differences between the concentrations of the two compounds is, on average, only a factor of two. To understand these unexpectedly high 2′*R*-OTA concentrations found in human blood, the affinity of this compound to the most abundant protein in human blood the human serum albumin (HSA) was studied and compared to that of OTA, which has a well-known high binding affinity. Using fluorescence spectroscopy, equilibrium dialysis, circular dichroism (CD), high performance affinity chromatography (HPAC), and molecular modelling experiments, the affinities of OTA and 2′*R*-OTA to HSA were determined and compared with each other. For the affinity of HSA towards OTA, a log*K* of 7.0–7.6 was calculated, while for its thermally produced isomer 2′*R*-OTA, a lower, but still high, log*K* of 6.2–6.4 was determined. The data of all experiments showed consistently that OTA has a higher affinity to HSA than 2′*R*-OTA. Thus, differences in the affinity to HSA cannot explain the relatively high levels of 2′*R*-OTA found in human blood samples.

## 1. Introduction

**Figure 1:**
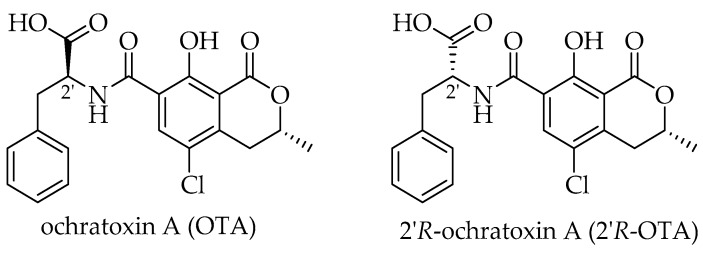
Molecular structures of OTA and its thermal isomer 2′*R*-OTA.

Ochratoxin A (OTA) is a toxic secondary metabolite produced by fungal species of the genus *Penicillium* and *Aspergillus* which can contaminate several agricultural commodities. Within the European Union (EU) cereals and cereal products such as pasta, bread and beer are the main sources, while roasted coffee only contributes ca. 10% to OTA exposure [1]. 2′*R*-Ochratoxin A (2′*R*-OTA, previously reported as 14*R*-ochratoxin A, Figure 1) is a diastereomer of OTA that is not produced by fungi but is formed by isomerization of OTA during thermal processing of contaminated food material. So far, 2′*R*-OTA has only been detectable in roasted coffee in concentrations of up to 0.63 µg/kg and on average ca. 20% rel. to OTA [2,3]. Considering the high contribution of cereals and cereal products, the total exposure to OTA is around fifty-fold higher compared to 2′*R*-OTA. In contrast to the exposure, both compounds could be found in the blood of coffee drinkers in a similar range (0.02–0.41 µg/L), with the 2′*R*-OTA concentration being on average half that of OTA. In certain cases, 2′*R*-OTA even exceeded the OTA concentrations [4,5].

Several studies have shown that OTA is nephrotoxic, hepatotoxic, immunotoxic and teratogenic. The International Agency for Research on Cancer (IARC) classified OTA as possibly carcinogenic to humans (group 2B) [6], and the Scientific Committee for Food of the European Commission suggests to reduce the OTA exposure to as low as reasonably achievable [1]. For the thermal degradation product 2′*R*-OTA, it is only known that it shows a ten-fold lower cytotoxic effect on IHKE and HepG2 cells compared to OTA [2,7].

The pharmacokinetics of OTA have been extensively studied in animals as well as in humans, as reviewed by Malir et al. [8,9,10]. For a single human volunteer, Studer-Rohr et al. demonstrated that OTA has a long biological half-life of approx. 35 days [11]. This long half-life and its accumulation in blood are attributed to the high affinity of OTA towards human serum albumin (HSA) [11]. Consequently, the excretion of OTA in human urine is very low [12].

For 2′*R*-OTA, no pharmacokinetic data are available. Yet, the high blood levels strongly indicate a long biological half-life and accumulation in blood.

HSA is the most abundant circulating protein in human blood, with a concentration in the g/L range. It is responsible for many processes, including maintaining the oncotic pressure, and HSA influences the fluid exchange between body compartments. HSA provides ligand binding capacity for drugs and xenobiotics, resulting in the important role of HSA in their pharmacokinetics [13]. Two binding sites for OTA were identified on HSA, and OTA interacts with albumin as a dianion [14]. Based on the study of Il’ichev et al., the binding site with the highest affinity to OTA is located in subdomain IIA (Sudlow’s Site I), while its second binding site exists in subdomain IIIA (Sudlow’s Site II) of HSA and has a 50-fold lower binding affinity [15]. More than 99.8% of OTA in human blood is non-covalently bound to HSA [9,11]. The HSA affinity to 2′*R*-OTA has not been studied, yet.

The aim of this study was to compare the binding affinities of OTA and 2′*R*-OTA to HSA in order to explain the high 2′*R*-OTA levels found in the blood of coffee drinkers. Therefore, different experimental approaches, namely equilibrium dialysis, fluorescence spectroscopy, circular dichroism (CD), high-performance affinity chromatography (HPAC), and molecular modeling studies were performed to provide a comprehensive dataset and understanding of the binding of 2′*R*-OTA to HSA and differences compared to OTA.

## 2. Results and Discussion

### 2.1. Dialysis of OTA and 2′R-OTA with HSA

In this experiment, an equilibrium dialysis cell consisting of two compartments separated by a 10 kDa cut-off dialysis membrane was applied. To determine the binding of OTA and 2′*R*-OTA to HSA, one compartment of the cell was loaded with a mixture of HSA and the respective ochratoxin in buffer, while the second compartment was solely loaded with buffer. As ochratoxins bound to HSA cannot permeate through the dialysis membrane but free ones can, an equilibrium between the concentrations of free ochratoxin in both compartments of the dialysis cell establishes. When the equilibrium is reached, analysis of the ochratoxin concentration in both dialysis chambers makes it possible to determine the ratio between unbound and HSA-bound ochratoxins.

**Figure 2:**
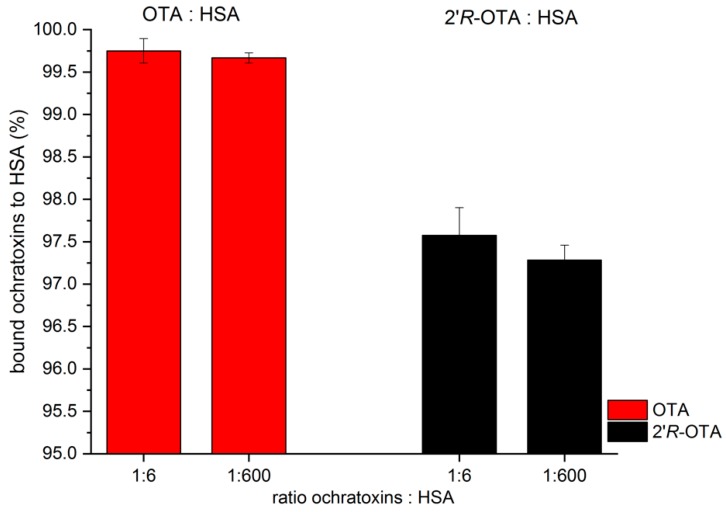
Percentage (±SD) of bound OTA (red bars) and 2′*R*-OTA (black bars) to a 6-fold and 600-fold excess HSA determined by equilibrium dialysis. Each experiment was performed in triplicate, and error bars represent ±SD.

This experiment was performed individually in triplicate for 25 nM OTA or 2′*R*-OTA using a 6-fold and 600-fold excess of HSA (0.15 µM and 150 µM), and the results are shown in Figure 2. After equilibration, 0.25 ± 0.15% free OTA were determined when a 6-fold excess of HSA was applied and 0.33 ± 0.06% of free OTA were determined with 600-fold excess of HSA. For 2′*R*-OTA, the respective values were 2.43 ± 0.33% for 6-fold excess of HSA and 2.72 ± 0.18% for 600-fold excess of HSA. No significant differences between the levels of free ochratoxin in the presence of different HSA concentrations (*p* < 0.05) were determined which is in good agreement with data from Il’ichev et al., where a ochratoxin:HSA ratio of 1:6 was found to be sufficient for establishing a steady concentration of free OTA [15]. OTA showed a significantly higher affinity towards HSA compared to 2′*R*-OTA (*p* ≤ 0.01, Figure 2). Over 99.6% of OTA but only 97.2% of 2′*R*-OTA were bound to HSA in the dialysis cell. These results indicate that the concentration of free 2′*R*-OTA is more than 10 times higher compared to OTA.

### 2.2. Fluorescence Spectroscopic Investigation of OTA and 2′R-OTA with HSA

**Figure 3:**
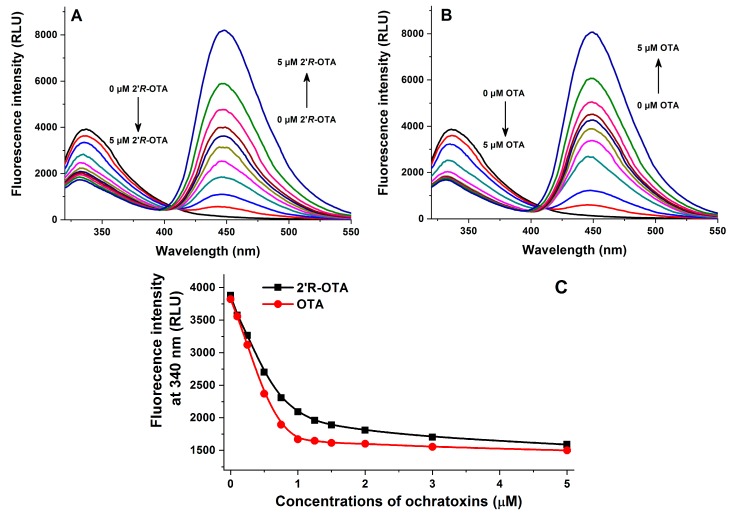
Fluorescence quenching of human serum albumin (HSA) by ochratoxins. Emission spectra of HSA (2 μM) in the presence of increasing 2′*R*-OTA (**A**: 0.0, 0.1, 0.25, 0.5, 0.75, 1.0, 1.25, 1.5, 2.0, 3.0, and 5.0 μM) and OTA (**B**: 0, 0.1, 0.25, 0.5, 0.75, 1.0, 1.25, 1.5, 2.0, 3.0, and 5.0 μM) concentrations in PBS (pH 7.4; λ_ex_ = 295 nm). Ochratoxin-induced decrease of the fluorescence emission signal of HSA at 340 nm (**C**) (*n* = 3). RLU: Relative Light Unit.

**Figure 4:**
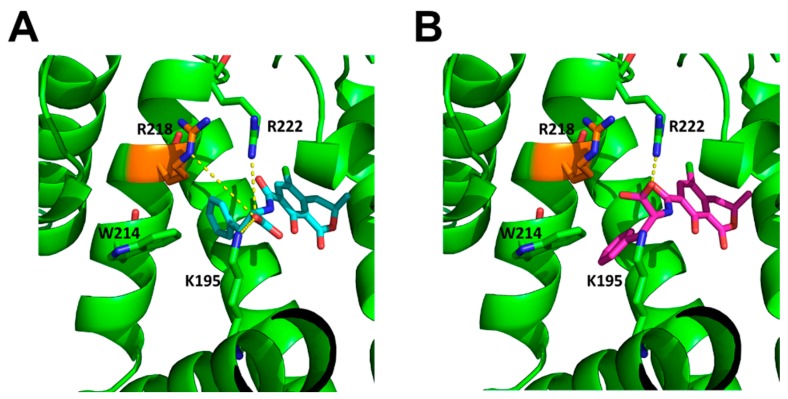
Structures of the aligned OTA (**A**, blue) and 2′*R*-OTA (**B**, magenta) in HSA (green). Important interacting residues are shown as sticks, salt-bridges are shown as yellow dashed lines. While OTA can form salt-bridge interactions with R218, R222, and K195, 2′*R*-OTA can only form these interactions with R222.

Fluorescence quenching of HSA by 2′*R*-OTA and OTA was investigated in the presence of 2 μM HSA and increasing concentrations of ochratoxins (0–5 μM) in PBS (pH 7.4). As Figure 3 demonstrates, even 0.1 μM of both mycotoxins induced a significant decrease of the fluorescence emission signal of HSA (λ_ex_ = 295 nm, λ_em_ = 340 nm), which suggests their potent interaction with the protein. The strong decrease in the fluorescence of HSA is presumably resulting from the quenching of the fluorescence of Trp-214 amino acid, which is located in subdomain IIA of HSA (see Figure 4). Therefore, it is reasonable to hypothesize that, similarly to OTA, the binding site of 2′*R*-OTA is located on Sudlow’s Site I (subdomain IIA) [15]. Figure 3C also highlights that OTA induces a steeper decrease of the fluorescence of HSA at 340 nm compared to 2′*R*-OTA which suggests the lower binding strength of 2′*R*-OTA-HSA than OTA-HSA. However, considering the significant quenching effect of 2′*R*-OTA on HSA, we can conclude the formation of very stable 2′*R*-OTA-HSA complexes. The second signal at 450 nm is derived from the intrinsic fluorescence emission of ochratoxins.

In the next series of experiments, increasing concentrations of HSA (0–5 μM) were added to standard levels of ochratoxins (1 μM both) in PBS (pH 7.4). Three different measurements were performed using these samples: (1) emission spectra were recorded using 295 nm as excitation wavelength (excitation of HSA); (2) emission spectra were recorded using 394 nm as excitation wavelength (excitation of HSA-bound ochratoxins); (3) fluorescence anisotropy values were determined using 394 and 447 nm as excitation and emission wavelengths, respectively (wavelength maxima of HSA-bound ochratoxins).

**Figure 5:**
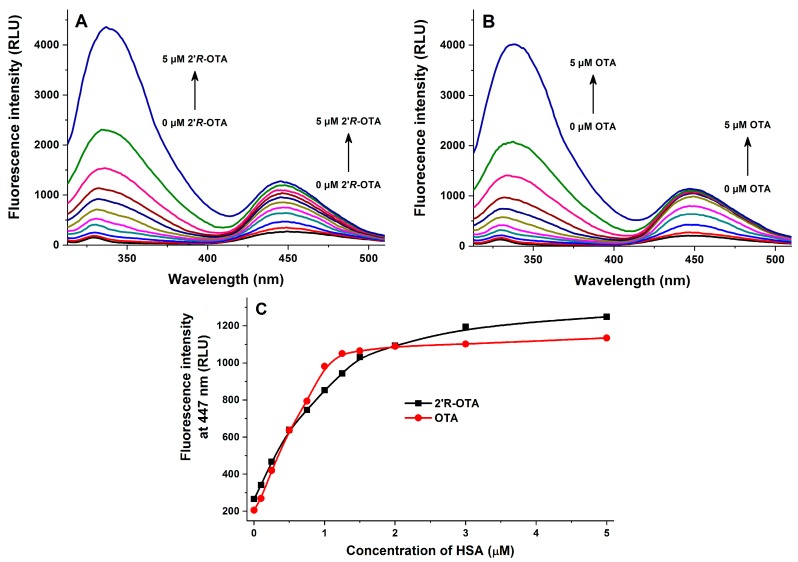
Fluorescence emission spectra of 2′*R*-OTA (**A**: 1 μM) and OTA (**B**: 1 μM) in the presence of increasing HSA concentrations (0, 0.1, 0.25, 0.5, 0.75, 1.0, 1.25, 1.5, 2.0, 3.0, and 5.0 μM) in PBS (pH 7.4) using 295 nm as excitation wavelength. Fluorescence emission intensities of 2′*R*-OTA and OTA at 447 nm (**C**) (*n* = 3).

In the first measurement, HSA was excited at 295 nm. The first signal at ca. 340 nm is derived from the fluorescence emission of increasing HSA concentrations (Figure 5). Furthermore, as the emission spectrum of HSA and the excitation spectrum of ochratoxins overlap and ochratoxins bind relatively close to the Trp-214 residue of HSA, the energy transfer between HSA and ochratoxins results in a significant emission of the HSA-bound mycotoxins at 447 nm. As Figure 5 demonstrates, the presence of increasing HSA concentrations led to the strong increase of emission intensities of ochratoxins at 447 nm, showing again their strong interaction with albumin. Under these circumstances, HSA alone did not give a significant emission at 447 nm. In agreement with quenching studies, this experiment suggests again the weaker interaction of 2′*R*-OTA with HSA compared to OTA.

**Figure 6:**
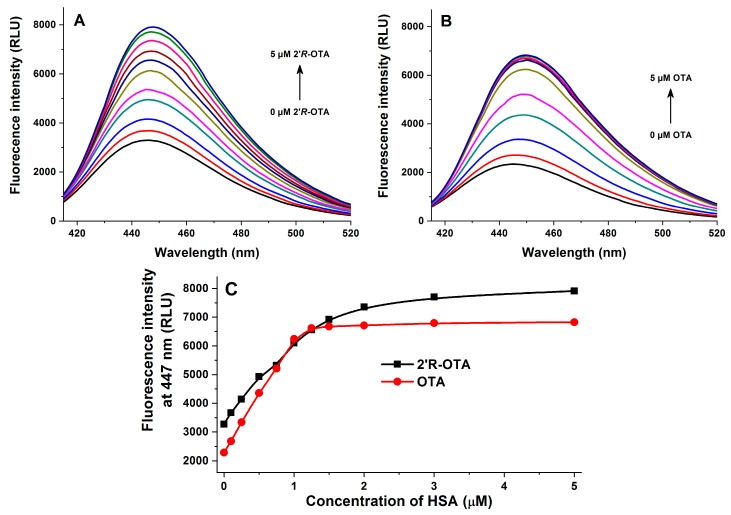
Fluorescence emission spectra of 2′*R*-OTA (**A**: 1 μM) and OTA (**B**: 1 μM) in the presence of increasing HSA concentrations (0, 0.1, 0.25, 0.5, 0.75, 1.0, 1.25, 1.5, 2.0, 3.0, and 5.0 μM) in PBS (pH 7.4) using 394 nm as excitation wavelength. Fluorescence emission intensities of 2′*R*-OTA and OTA at 447 nm (**C**) (*n* = 3).

In the second experiment, ochratoxins and ochratoxin-HSA complexes were excited at 394 nm. Fluorescence emission intensities of ochratoxins are significantly increased during their complex formation with HSA [16,17], while the emission wavelength maxima of free and HSA-bound ochratoxins are also similar (Figure 6). Therefore, the gradual elevation of the fluorescence of ochratoxins in the presence of increasing HSA concentrations is a result of the increased presence of HSA-bound ochratoxins and the decreased amount of free ochratoxins in the samples. Under these circumstances, HSA alone did not show an emission at 447 nm. The observation that OTA reached its maximal emission in the presence of 1.25–1.5 μM HSA, while *2′R*-OTA showed its maximum in the presence of approximately 3–5 μM HSA suggests again the formation of more stable complexes of HSA with OTA.

**Figure 7:**
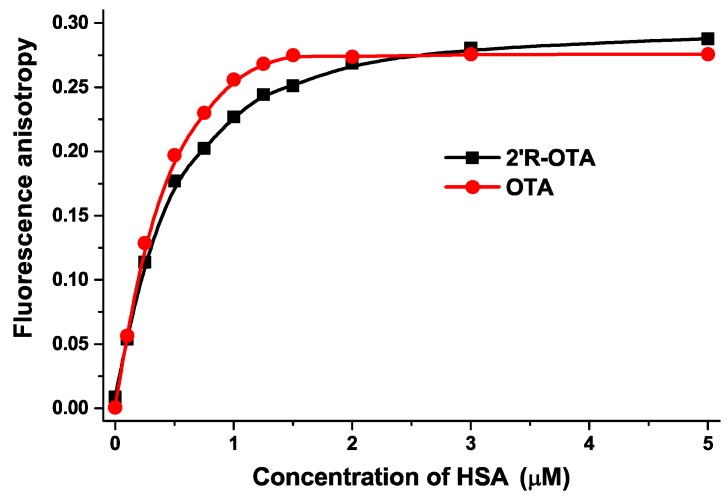
Fluorescence anisotropy values of 2′*R*-OTA and OTA (both 1 μM) in the presence of increasing HSA concentrations (0, 0.1, 0.25, 0.5, 0.75, 1.0, 1.25, 1.5, 2.0, 3.0, and 5.0 μM) in PBS (pH 7.4; λ_ex_ = 394 nm, λ_em_ = 447 nm) (*n* = 3).

In the third experiment, fluorescence anisotropy values of ochratoxins were determined in the presence of increasing albumin concentrations (λ_ex_ = 394 nm, λ_em_ = 447 nm) (Figure 7). Since anisotropy characterizes the rotational freedom of fluorophores, the increased anisotropy values in the presence of HSA suggest the decreased rotational freedom of ochratoxins which obviously resulted from their complex formation with the macromolecule HSA. Fluorescence anisotropy also confirmed the results of the previously applied intensity-based methods: again, a weaker interaction of 2′*R*-OTA was observed with HSA compared to OTA.

Based on fluorescence spectroscopic investigation of 2′*R*-OTA-HSA complex formation (see in Figure 3, Figure 5, Figure 6 and Figure 7), the binding constant of the 2′*R*-OTA-HSA complex was determined (see details in Section 4.5). As Table 1 demonstrates, 2′*R*-OTA forms very stable complexes with HSA (binding constant (*K*) of ~10^6^ L/mol). On the other hand, this complex stability is approximately one magnitude lower compared to the binding constant of the OTA-HSA complex (~10^7^ L/mol) [16,17,18].

### 2.3. High-Performance Affinity Chromatography of HSA with OTA and 2′R-OTA

A high-performance affinity chromatography (HPAC) column coated with immobilized HSA (Chiralpak HSA HPLC Column (2.0 × 50 mm, 5 µm)) in combination with mass spectrometric detection using selected ion monitoring (SIM) was used to compare the affinity of both ochratoxins to HSA. The different affinities towards the immobilized HSA resulted in different retention times; the higher the affinity of the compound to the protein, the longer the retention time. Singh et al. showed that the HSA-HPAC chromatographic method is a suitable tool to determine the protein binding of different compounds [19]. Both analytes had a very long retention time compared to other compounds [19]. OTA had a significantly longer retention time than 2′*R*-OTA under these conditions (Table 2). The 3.2 min difference in the retention time clearly indicates that the non-covalent binding of OTA to HSA is stronger than of 2′*R*-OTA.

### 2.4. Circular Dichroism (CD) of HSA with OTA and 2′R-OTA

**Figure 8:**
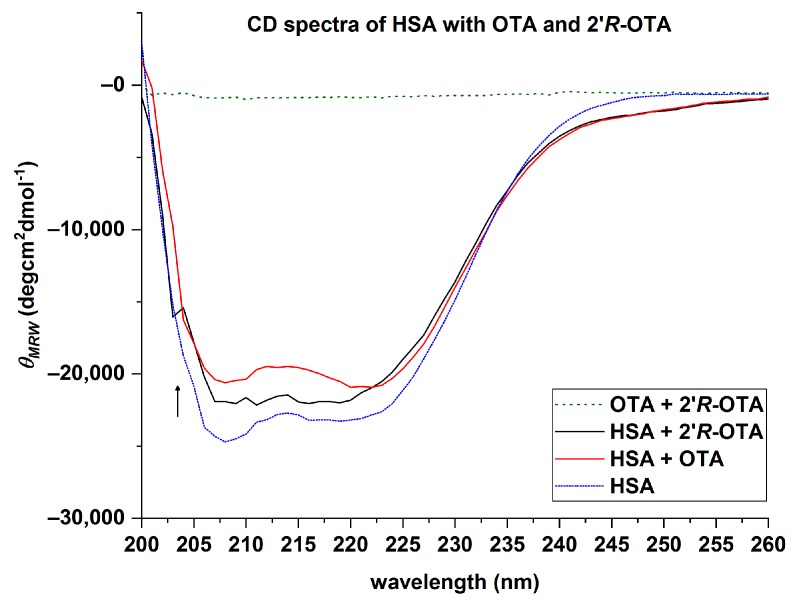
Average CD spectra of 0.7 µM HSA, OTA-HSA, 2′*R*-OTA-HSA complex, 14 µM OTA and 2′*R*-OTA.

To recognize changes in the secondary structure of HSA in the presence of ochratoxins, CD-spectra were recorded from 200 to 260 nm. To ensure that all HSA molecules (0.7 µM) are bound to the ochratoxins, a 20-fold excess of OTA and 2′*R*-OTA (14 µM) was incubated with the protein in PBS (pH 7.6). The mean residue ellipticity (*θ_MRE_*) of HSA at the characteristic wavelength minima of 208 and 222 nm indicated that the native protein is predominantly an α-helix (Figure 8, blue line). The amino acids of the protein are responsible for the α-helix characteristic minima [20] as the ochratoxins did not show a signal between 200 and 260 nm. In the presence of OTA and 2′*R*-OTA, a slight increase of *θ_MRE_* was observed at these characteristic wavelengths. This indicates that both ochratoxins lead to a change in the secondary structure of the native HSA, resulting in a decrease of the α-helicity (Figure 8). The native HSA had an α-helix percentage of about 67% (Table 3). As summarized in Table 3, OTA had a stronger influence on the secondary structure of HSA compared to 2′*R*-OTA.

**Figure 9:**
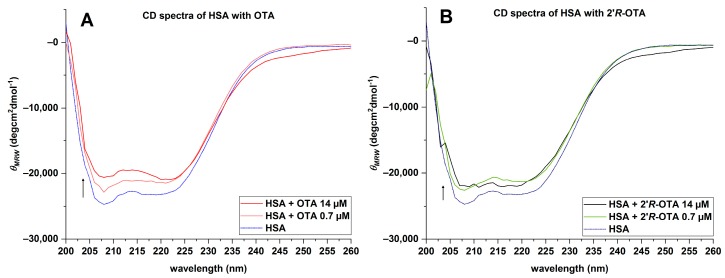
Average CD spectra of HSA at different OTA (**A**: 0.7–14 µM) and 2′*R*-OTA (**B**: 0.7–14 µM) concentrations.

In addition, HSA was incubated with equimolar concentrations of ochratoxins under the same conditions. For OTA it is known that HSA has two different binding sites, but that one is preferred. In the CD spectra of HSA with two different concentrations of OTA a stepwise increase of the *θ_MRE_* was noticed, as demonstrated in Figure 9A. For HSA with two different concentrations of 2′*R*-OTA, this effect was not observed. With an increase of the 2′*R*-OTA concentration, no change in the CD spectra was obtained (Figure 9B).

### 2.5. Molecular Modelling Studies of OTA and 2′R-OTA with HSA

The HSA complexes with OTA and 2′*R*-OTA were generated by overlaying the ligands onto the co-crystallized warfarin of the HSA crystal structure (PDB-ID: 1H9Z) using the largest common substructure (Supplementary Materials Figure S1). Subsequently, molecular dynamics (MD)-simulations of the HSA-OTA and HSA-2′*R*-OTA complexes were performed. From the MD trajectories, complex configurations were extracted and used to compute endpoint effective binding energies, employing the Molecular Mechanics-Poisson-Boltzmann Surface Area (MM-PBSA) approach. This resulted in an effective binding energy of −32.28 ± 4.49 kcal mol^−1^ (mean ± SD) for OTA to HSA and −20.09 ± 4.60 kcal mol^−1^ (mean ± SD) for 2′*R*-OTA to HSA, indicating a stronger binding affinity of OTA to HSA. Please note that configurational entropy differences were neglected here (see Methods section for details), such that the difference in the binding free energies of the diastereomers may be smaller due to enthalpy-entropy compensation effects. The difference in effective binding energy can be linked to OTA forming salt-bridges with its carboxylic acid group to R218 (80.7% occupancy over the trajectory), R222 (98.4%), and K195 (5.2%) of HSA (Figure 4A), while 2′*R*-OTA can only form salt-bridges with R222 (50.5%) due to the different configuration (Figure 4B).

## 3. Conclusions

This is the first study investigating the affinity of the mycotoxin 2′*R*-OTA towards the protein HSA. Therefore, different experimental and theoretical approaches were performed. The results of all experiments are consistent and show that HSA has a high binding affinity towards both mycotoxins although the affinity of HSA to OTA is clearly stronger than to 2′*R*-OTA.

The dialysis experiment demonstrated that over 99.6% of OTA and 97.2% of 2′*R*-OTA are bound to HSA. The results concerning OTA are in agreement with literature data in which it was shown that only 0.2% of unbound OTA circulate in human blood [11]. In the case of 2′*R*-OTA, the percentage of unbound molecules in human blood is not known. However, the dialysis experiments with HSA shows that solely binding to this protein would result in approx. 10 times more unbound 2′*R*-OTA compared to OTA. In this case, it could be expected that excretion of 2′*R*-OTA compared to OTA is faster, and thus a shorter biological half-life would occur. Fluorescence spectroscopic studies yielded decimal logarithmic values of the binding constant for 2′*R*-OTA of 6.2–6.4 and approximately 10-fold higher log*K* values for OTA (7.0–7.6) [16,17,18]. By HPAC experiments, a weaker interaction of 2′*R*-OTA with immobilized HSA compared to OTA was shown as well. The CD spectra of HSA with both ochratoxins revealed a change in the secondary structure of the protein, whereby OTA had a stronger influence than 2′*R*-OTA in decreasing the α-helicity. End-point effective binding energy computations of the HSA-ochratoxin complexes demonstrated a higher effective binding energy for HSA-OTA than for HSA-2′*R*-OTA.

These results were somewhat unexpected, as the stronger binding affinity of HSA to OTA compared to 2′*R*-OTA, which was shown in all experiments, cannot explain the observed difference in exposure and presence in human blood of the two ochratoxins. As described above, the highest OTA:2′*R*-OTA ratio in food samples is 4:1 and in human plasma samples 1:1; however, the observed differences in HSA binding seems to be not responsible for this effect. Further research studies have to be done to understand this discrepancy. Possible reasons for the accumulation of 2′*R*-OTA in human blood might be another human blood protein with a very high affinity to 2′*R*-OTA or a higher bioavailability of 2′*R*-OTA compared to OTA or differences in the metabolism or transport in human tissues.

## 4. Materials and Methods

### 4.1. Reagents

Methanol and acetonitrile were obtained in gradient grade from Fisher Scientific (Schwerte, Germany). Purified water of ASTM type 1 was prepared with a Purelab Flex 2 system from Veolia Water Technologies (Celle, Germany). Human serum albumin ≥99% purity was purchased from Merck (Steinheim, Germany) and further chemicals were purchased either for Merck or Roth (Karlsruhe, Germany) in analytical grade.

For circular dichroism measurements a 30 mM PBS (pH 7.6) containing 4.4 g Na_2_HPO_4_ and 0.6 g KH_2_PO_4_ in 1 L purified water and for the dialysis experiments a 10 mM PBS (pH 7.4) containing 1.2 g Na_2_HPO_4_ and 0.58 g NaCl in 1 L purified water was used. For the ammonium acetate puffer (50 mM, pH 7.4) 3.8 g ammonium acetate was dissolved in 1 L purified water.

### 4.2. Biosynthesis of Standards

OTA was isolated from cultures of *Aspergillus westerdijkiae* BFE 1115, kindly provided by the Max Rubner Institute (Karlsruhe, Germany), which were activated for 2 days in liquid potato dextrose broth at room temperature and then incubated for two weeks at 27 °C on autoclaved durum wheat. Therefore 50 jars (200 mL) filled with 40 g durum wheat containing additional 62.5% water (*w*/*w*) and 2.5% sodium chloride (*w*/*w*) were capped with Magenta B-caps (Merck), sterilized for 30 min at 121 °C and inoculated with 1 mL of the liquid *A. westerdijkiae* potato dextrose culture. After incubation for two weeks at 27 °C, the cultures of every jar were three times extracted with a total of 100 mL *t*BME/formic acid (99/1, *v*/*v*) for 2 h on a laboratory shaker at 250 rpm. Extracts were filtered, combined and concentrated to a volume of approximately 700 mL using a rotary evaporator. The pH of the extract was set to pH 7 with 1 M NaOH and the solution purified by liquid-liquid extraction with 3% NaHCO_3_ (3 × 500 mL). While the organic phase was discarded, the aqueous phase was adjusted to pH 2 with concentrated formic acid (FA) and re-extracted with *t*BME (3 × 700 mL). The combined organic phases were subsequently evaporated to dryness. For further purification, the residue was dissolved in toluene/*t*BME/FA (8/1.5/0.5, *v*/*v*/*v*) and subjected to silica column chromatography with toluene/*t*BME/FA (8/1.5/0.5, *v*/*v*/*v*) as solvent. OTA was detected by its fluorescence under UV-light, the respective fractions were combined and evaporated. Multiple crystallization from xylene/hexane (7/3, *v*/*v*) yielded 330 mg OTA with a purity >99% determined by HPLC-UV (220 nm). The structure of OTA was confirmed by ^1^H-NMR-spectroscopy (see Supplementary Materials Figures S2 and S4).

The isomerization of OTA to 2′*R*-OTA and isolation of 2′*R*-OTA was performed as described by Cramer et al. [2]. Therefore, 20 mg OTA were heated at 200 °C for 20 min and 10 mg 2′*R*-OTA was separated from the remaining OTA by preparative HPLC-UV with a purity of >99%. The structure was characterized by NMR and was in agreement with literature data (see Supplementary Materials Figures S3 and S4).

### 4.3. Dialysis Experiments

For the dialysis experiments a micro biochem dialyzing chamber made of PTFE for equilibrium dialysis (2 × 500 µL volume) and a cellulose-membrane with 10 kDa cut-off both from Reichelt Chemietechnik (Heidelberg, Germany) was used. Ochratoxins were incubated with HSA for 30 min at room temperature while shaking before the dialysis experiment. The equilibrium dialysis cell had two compartments which were separated by a semipermeable membrane with a 10 kDa cut-off. Dialysis was performed in two independent experiments either with 25 nM OTA or 2′*R*-OTA (4.03 ng/0.4 mL) and 0.15–15 µM HSA (3.96–369 µg/0.4 mL) in PBS in one compartment (10 mM, pH 7.4) against PBS in the other compartment for 5.5 h at 37 °C. The ochratoxins could pass through the membrane while HSA (67 kDa) could not pass and remains in one compartment. After 5.5 h equilibration an aliquot of 100 µL of both compartments were analyzed by HPLC-MS/MS. The score of OTA in the dialyzing compartment without HSA corresponds to the free OTA while the score of OTA in the dialyzing compartment with HSA corresponds to bound and free OTA. For analysis, HSA was precipitated by addition of 100 µL acetonitrile. The samples were centrifuged at 2500× *g* and 50 µL of the supernatant were diluted with 50 µL water and analyzed with HPLC-MS/MS. To get the timepoint when the equilibrium is reached and to proof the recovery, the two compounds were dialyzed in PBS without HSA and an aliquot on both sites were taken at different timepoints and measured with HPLC-MS/MS. Prior to the final experiment, the equilibration time of the dialysis cell was measured. Therefore, both compartments were loaded with buffer and OTA was added to one compartment. After 1, 2, 3, 4, 5, 8, 12, and 24 h, OTA concentrations were measured in both compartments. After 5 h, equilibrium was reached, and no further change of the OTA concentrations was observed. For all further experiments, equilibration was expected to be reached after 5.5 h. Recoveries were, in total, between 101% and 107% for OTA and 2′*R*-OTA. For the calculation of the recovery the sample of the OTA, 2′*R*-OTA and a combination of both ochratoxins in PBS buffer were measured with HPLC-MS/MS before being applied in the equilibrium dialyzing chamber and set to 100%.

Statistical evaluation was done by a one-way ANOVA and Tukey’s multiple-comparison test using OriginPro 2016G SR1. Signification levels (*p* ≤ 0.01 and *p* ≤ 0.05) are reported in the text.

### 4.4. HPLC-MS/MS Parameters for the Dialysis Experiments

The quantification of OTA and 2′*R*-OTA was carried out based on external calibration on a 1260 Infinity LC system (Agilent, Waldbronn, Germany) coupled to a QTRAP 6500 mass spectrometer (SCIEX, Darmstadt, Germany) that was operated in selected monitoring mode (SRM). Analyst 1.6.2 software was used for data acquisition and quantification. Electrospray ionization (ESI) was used for the ionization with +5.5 kV in positive mode and with following settings: source temperature 450 °C, curtain gas 40 psi, nebulizer gas 45 °C, heater gas 55 psi. Three SRM transitions were used for the quantification and the identification of both analytes are shown in Table 4.

For the separation, a Nucleodur C_18_ Gravity SB column (2.0 × 100 mm, 3 µm) (Macherey-Nagel, Düren, Germany) was used with a binary gradient of acetonitrile containing 2% acetic acid as solvent A and water containing 0.1% acetic acid as solvent B with the following conditions: 0 min 20% A (0.4 mL/min), 2 min 20% A (0.4 mL/min), 4.5 min 50% A (0.4 mL/min), 7 min 100% A (0.5 mL/min), 9 min 100% A (0.5 mL/min), 9.1 min 20% A (0.4 mL/min), 12 min 20% A (0.4 mL/min). OTA and 2′*R*-OTA were baseline separated with the retention time of 6.6 min for OTA and 6.8 min for 2′*R*-OTA (see Figure S5).

The injection volume was 30 µL of each sample, and oven temperature was set to 40 °C.

### 4.5. Fluorescence Spectroscopic Measurments

Fluorescent measurements were performed employing a Hitachi F-4500 fluorescence spectrophotometer (Tokyo, Japan). In order to mimic extracellular physiological conditions, mycotoxin-albumin interactions were studied in PBS (pH 7.4). All measurements were carried out at 25 °C in the presence of air.

Complex formation of 2′*R*-OTA with HSA was examined applying the Stern-Volmer equation:

(1) $$\frac{I_{0}}{I}=1+K_{SV}\times [Q]$$

where *I* and *I*_0_ denote the fluorescence intensities of HSA in the absence and presence of 2′*R*-OTA, respectively. *K_SV_* (with the unit of L/mol) is the Stern-Volmer quenching constant and [*Q*] is the molar concentration of the quencher (2′*R*-OTA). In order to eliminate the inner-filter effect, UV-Vis spectrum of 2′*R*-OTA was recorded using a Specord Plus 210 spectrophotometer (Analytic Jena AG, Jena, Germany), and fluorescence intensities were corrected applying the following equation [21]:

(2) $$I_{\mathrm{cor}}=I_{\mathrm{obs}}\times e^{(A_{\mathrm{ex}}+A_{\mathrm{em}})/2}$$

where *I_cor_* and *I_obs_* are the corrected and observed fluorescence emission intensities, respectively; while *A_ex_* and *A_em_* are the absorption values of 2′*R*-OTA at 295 and 340 nm, respectively.

Overall and stepwise binding constants were calculated by non-linear fitting using the fluorescence emission data obtained for all the performed experiments (quenching of the fluorescence of HSA by 2′*R*-OTA, fluorescence enhancement induced by the energy transfer between HSA and 2′*R*-OTA, and fluorescence enhancement of 2′*R*-OTA by HSA) with the Hyperquad2006 program package. To calculate the stability constants associated with the complex formation between HSA and 2′*R*-OTA, the following equations are implemented in the Hyperquad code [18,22]:

(3) $$p\mathrm{HSA}+q\mathrm{OTA}↔{\mathrm{HSA}}_{p}{\mathrm{OTA}}_{q}$$

(4) $$\beta_{\mathrm{pq}}=\frac{\left[{\mathrm{HSA}}_{p}{\mathrm{OTA}}_{q}\right]}{{\left[\mathrm{HSA}\right]}^{p}{\left[\mathrm{OTA}\right]}^{q}}$$

where *p* and *q* are the coefficients which indicate the stoichiometry associated with the possible equilibrium in the solution. In the Hyperquad2006 computer fitting program, all equilibrium constants are defined as overall binding constants.

(5) $$\mathrm{HSA}+\mathrm{OTA}↔\mathrm{HSA}\;\mathrm{OTA}\;\beta_{1}=\frac{\left[\mathrm{HSAOTA}\right]}{\left[\mathrm{HSA}\right]\left[\mathrm{OTA}\right]}$$

(6) $$\mathrm{HSA}+q\mathrm{OTA}↔{\mathrm{HSA}\;\mathrm{OTA}}_{q}\;\beta_{q}=\frac{\left[{\mathrm{HSA}\;\mathrm{OTA}}_{q}\right]}{\left[\mathrm{HSA}\right]{\left[\mathrm{OTA}\right]}^{q}}$$

The relationship between the overall binding constants and the stepwise binding constants calculated by the Hyperquad is the following.

(7) $$\beta_{1}=K_{1};\;\beta_{q}=\;K_{1}\times K_{2}\ldots \times K_{q}$$

The stoichiometry and binding constant of 2′*R*-OTA-HSA complex were determined by the model associated with the lowest standard deviation.

Fluorescence anisotropy (*r*) data were determined using the following equation:

(8) $$r=\frac{\left(I_{\mathrm{VV}}-G\times I_{\mathrm{VH}}\right)}{\left(I_{\mathrm{VV}}+2\times {G\times I}_{\mathrm{VH}}\right)}$$

where *I_VV_* and *I_VH_* are fluorescence emission intensities measured in vertical position of polarizer at pre-sample site and at vertical and horizontal position of the post-sample polarizer, respectively, while *G* is the instrumental factor. Considering the additive behavior of anisotropy, the following equation can be described:

(9) $$r=f_{f}\times r_{f}+f_{b}\times r_{b}$$

where *f_f_* and *f_b_* are the free and HSA-bound fractions of 2′*R*-OTA in the solution, respectively, while *r_f_* and *r_b_* are the anisotropies of free and HSA-bound 2′*R*-OTA, respectively. The free HSA-bound fractions of 2′*R*-OTA can be described from the rearrangement of Equation (9).

(10) $$f_{f}=\frac{\left(r-r_{b}\right)}{\left(r_{f}-r_{b}\right)}$$

(11) $$f_{b}=1-f_{f}$$

Furthermore, assuming 1:1 stoichiometry of complex formation as well as through the application of Equations (10) and (11), the binding constant (*K*) can be expressed with the following equation:

(12) $$K=\frac{f_{b}/\theta }{f_{f}\times [\mathrm{HSA}]}$$

where [*HSA*] is the albumin concentration, and *θ* is the change in quantum yield (*I_b_* and *I_f_* are the fluorescence emission intensities of HSA-bound and free 2′*R*-OTA, respectively).

(13) $$\theta =\frac{I_{b}}{I_{f}}$$

### 4.6. HPAC-MS Measurments

An Agilent 1290 Infinity LC system (Agilent, Waldbronn, Germany) was coupled to an Agilent 6120 MS Quadrupole system in ESI mode, which was used in the positive selected ion monitoring (SIM) mode with *m*/*z* 404. For the high-performance affinity chromatography (HPAC) a Chiralpak HSA HPLC column (2.0 × 50 mm, 5 µm) (Chiral Technologies Europe SAS, Illkrich Cedex, France) was used with an isocratic gradient of 80% ammonium acetate buffer (50 mM, pH 7.4) and 20% isopropanol with a flow rate of 0.25 mL/min. 5 µL of a 10 µM OTA and 2′*R*-OTA solution in eluent ammonium acetate puffer and isopropanol (8/2 (*v*/*v*)) was injected.

### 4.7. Circular Dichroism Measurements

The circular dichroism spectra were measured with a Jasco J-600 CD spectrometer (Jasco, Groß-Umstadt, Germany) at room temperature using a 1 cm cell. The spectra were record between 200 and 260 nm with a step size of 1 nm, bandwidth of 1 nm, speed of 100 nm/min and an average time of 0.5 s. Five measurements of each sample were accumulated and averaged without using the smoothing function. Different OTA and 2′*R*-OTA concentrations (0.7–14 µM) were incubated in duplicate with 0.7 µM HSA in PBS (30 mM, pH 7.6) for 5 h at room temperature while shaking. For all experiments, one HSA solution with a concentration of 0.7 µM in PBS (10 mM, pH 7.4) determined by Bradford assay was used. For converting the observed ellipticity (*θ_obs_*) to the mean residue ellipticity (*θ_MRE_*) Equation (14) was used

(14) $$\theta_{\mathrm{MRE}}=\frac{\theta_{\mathrm{obs}}}{10\times C_{p}\times n\times l}$$

where *C_p_* is the protein concentration (6.7 × 10^−7^ M), *n* is the number of amino acids of HSA (584) and l is the length of the cuvette (1 cm). For the calculation of the α-helix structure Equation (15)

(15) $$\alpha -\mathrm{helix}\;(\%)=\frac{-\theta_{\mathrm{MRE}}-4000}{33000-4000}\times 100$$

and the software K2D3 were utilized [20,23,24]. For the K2D3 software the $\theta_{\mathrm{MRE}}$ from 200 to 240 nm and the protein size of 584 amino acids were applied.

### 4.8. Molecular Modeling Studies

To generate the complexes, OTA and 2′*R*-OTA were aligned onto the co-crystallized structure of Warfarin in human serum albumin (HSA; PDB-ID: 1H9Z) via core matching using Moloc [25] and energetically relaxed in the binding pocket. Subsequently, 100 ns of all-atom molecular dynamics (MD)-simulations of each HSA-Ochratoxin A diastereomer complex were performed. For this, the protonation states of HSA were assigned with PROPKA [26] according to pH 7.4, while OTA and 2′*R*-OTA were modeled as dianions according to pKa values reported in PubChem (PubChem CID 442530). Ligand charges were determined via the RESP procedure [27]. The complexes were protonated with PROPKA [26] according to pH 7.4, neutralized by adding counterions, and solvated in an octahedral box of TIP3P water [28] with a minimal water shell of 12 Å around the solute. The Amber16 package of molecular simulation software [29] with ff14SB and GAFF [30] force fields were used to perform the MD simulations. To cope with long-range interactions, the “Particle Mesh Ewald” method [31] was used; the SHAKE algorithm [32] was applied to bonds involving hydrogen atoms. The time step for all MD simulations was 2 fs with a direct-space, non-bonded cut-off of 8 Å.

At the beginning, 17,500 steps of steepest decent and conjugate gradient minimization were performed; during 2500, 10,000, and 5000 steps, positional harmonic restraints with force constants of 25 kcal mol^−1^ Å^−2^, 5 kcal mol^−1^ Å^−2^, and zero, respectively, were applied to the solute atoms. Thereafter, 50 ps of NVT-MD (MD simulations with a constant number of particles, volume, and temperature) were conducted to heat up the system to 100 K, followed by 300 ps of NPT-MD (MD simulations with a constant number of particles, pressure, and temperature) to adjust the density of the simulation box to a pressure of 1 atm and to heat the system to 300 K. During these steps, a harmonic potential with a force constant of 10 kcal mol^−1^ Å^−2^ was applied to the solute atoms. As the final step in thermalization, 300 ps of NVT-MD simulations were performed while gradually reducing the restraint forces on the solute atoms to zero within the first 100 ps of this step. Afterwards, a production run of NVT-MD simulation with 100 ns length was performed. NVT-MD simulations are the standard procedure for non-membrane MD simulations using the free energy workflow FEW [33]. Compared to NVE simulations, the temperature is kept constant in NVT simulations, which agrees with isothermal conditions in laboratories. In contrast to NPT simulations, the volume, but not the pressure, is kept constant in NVT simulations. First, this renders it unnecessary to use a barostat, which, if not appropriate, may lead to the generation of a not well-defined thermodynamic ensemble. Second, in condensed phase simulations such as ours, volume fluctuations are generally small, such that the condition of keeping the volume constant is a good approximation.

Subsequently, the free energy workflow FEW [33] was used to calculate effective binding energies of the complexes. To do so, we extracted snapshots every 20 ps from the MD trajectories, resulting in 5000 snapshots per complex, which were stripped off water and ions. The molecular mechanics and Poisson-Boltzmann surface-area calculations were performed with the mmpbsa.pl module [34] of Amber16 [29]. The one-trajectory approach with Parse radii [35], dielectric constants of 1 and 80 for the solute and the solvent, respectively, and an ionic strength of 0.15 mM was used to calculate the effective energies. While the one-trajectory approach neglects energetic effects due to conformational changes upon binding, it generally results in lower statistical uncertainties [36]. Contributions due to changes in the configurational entropy of the ligand or the receptor upon complex formation were neglected, too, in order to avoid introducing additional uncertainty in the computations [37,38,39].

## Figures and Tables

**Table 1:** Decimal logarithmic values of Stern-Volmer quenching constant and binding constant of 2′*R*-OTA-HSA complex (log*K* value of OTA-HSA complex was approximately 7.0–7.6) (*n* = 3).

Complex	log*K_SV_* (SV-Plot, Figure 3)	log*K* (Hyperquad, Figure 3)	log*K* (Hyperquad, Figure 5)	log*K* (Hyperquad, Figure 6)	log*K* (Anisotropy, Figure 7)
2′*R*-OTA-HSA	5.94 ± 0.02	6.23 ± 0.01	6.36 ± 0.06	6.40 ± 0.24	6.18 ± 0.01

**Table 2:** Retention times of OTA and 2′*R*-OTA on the HSA-HPAC column.

Compound	Retention Time ± SD (min) ^1^
OTA	20.1 ± 0.9
2′*R*-OTA	16.9 ± 0.2

^1^ mean of three repetitions.

**Table 3:** Mean residue ellipticity and α-helix contents of 0.7 µM HSA in presence of 14 µM OTA and 14 µM 2′*R*-OTA in PBS. α-helix percentage calculated with Equation (15) * and with K2D3 software **.

HSA + Ochratoxin (Ratio)	*θ_MRE_* (×10^2^) (deg × cm^2^ × dmol^−1^)		α-Helix * (%)		α-Helix ** (%)	Rel. Differences to HSA
	208 nm	222 nm	208 nm	222 nm		
HSA	−247	−228	71.4	65.0	66.9	-
HSA + 2′*R*-OTA (1:20)	−220	−210	61.8	58.5	65.3	2–13%
HSA + OTA (1:20)	−206	−209	57.3	58.3	61.5	8–20%

**Table 4:** Detailed single reaction monitoring (SRM) parameters for both analytes OTA and 2′*R*-OTA.

Q1 Mass (*m*/*z*)	Q3 Mass (*m*/*z*)	Transition Time (ms)	DP (V)	CE (V)	EP (V)
404.1	239.0 (quantifier)	100	+70	+31	+10
404.1	221.1 (qualifier)	100	+70	+47	+10
404.1	102.0 (qualifier)	100	+77	+88	+10

## Supplementary Materials

The Supplementary Materials are available online at http://www.mdpi.com/2072-6651/10/7/256/s1. Figure S1: Structural alignment of the ligands, Figure S2: ^1^H-NMR (400 MHz, Benzene-*d*_6_) spectra of ochratoxin A, Figure S3: ^1^H-NMR (400 MHz, Benzene-*d*_6_) spectra of 2′*R*-ochratoxin A, Figure S4: Molecular structure of the ochratoxins OTA and 2′*R*-OTA with labeled atoms, Figure S5: HPLC-MS/MS chromatogram of OTA and 2′*R*-OTA with the three transitions from the dialysis chamber without HSA after 5.5 h.
