# Supplementary material for: Interaction of Ochratoxin A and Its Thermal Degradation Product 2′R-Ochratoxin A with Human Serum Albumin
Source: Toxins (Basel). 2018 Jun 22;10(7):256. doi: 10.3390/toxins10070256 (PMC6070976; doi:10.3390/toxins10070256)
Supplement: Supplementary file 1 [file toxins-10-00256-s001.pdf]

Supplementary Materials: Interaction of Ochratoxin A and its thermal degradation product 2′*R*-Ochratoxin A with Human Serum Albumin

Franziska Sueck, Miklós Poór, Zelma Faisal, Christoph G. W. Gertzen, Benedikt Cramer, Beáta Lemli, Sándor Kunsági-Máté, Holger Gohlke and Hans-Ulrich Humpf

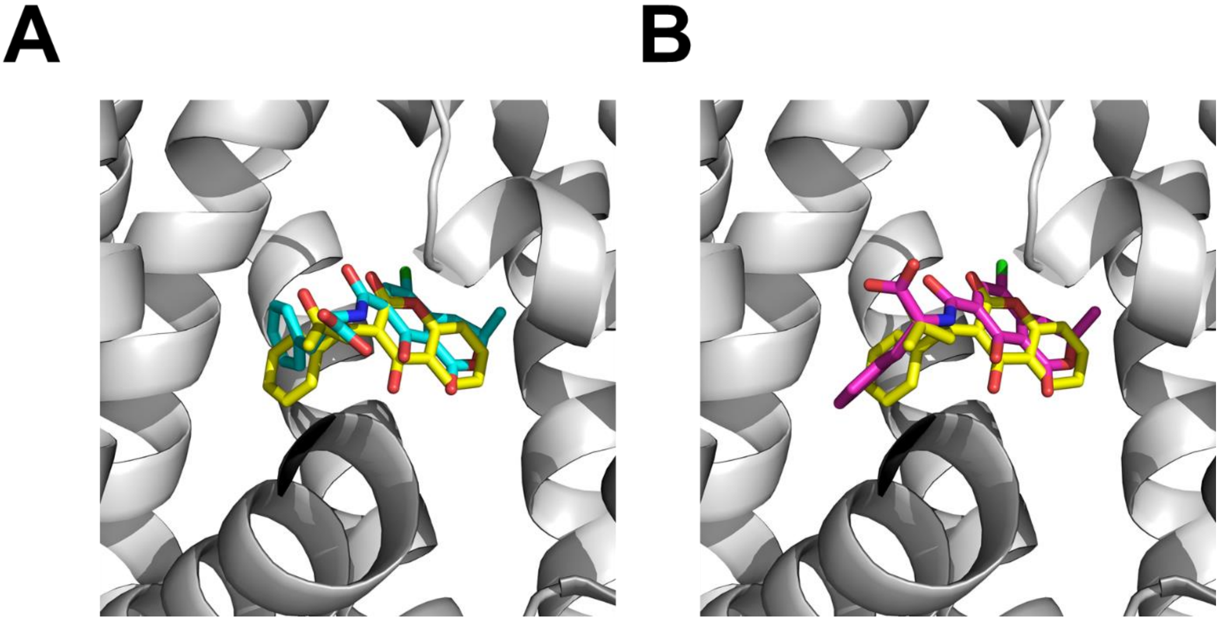

Figure S1. Structural alignment of the ligands. Comparison of OTA (**A**, blue) and 2′*R*-OTA (**B**, magenta) aligned onto Warfarin (yellow) in the crystal structure of the complex with HSA (grey, cartoon representation) (PDB ID: 1H9Z) after energetic relaxation of OTA and 2′*R*-OTA in the presence of HSA.

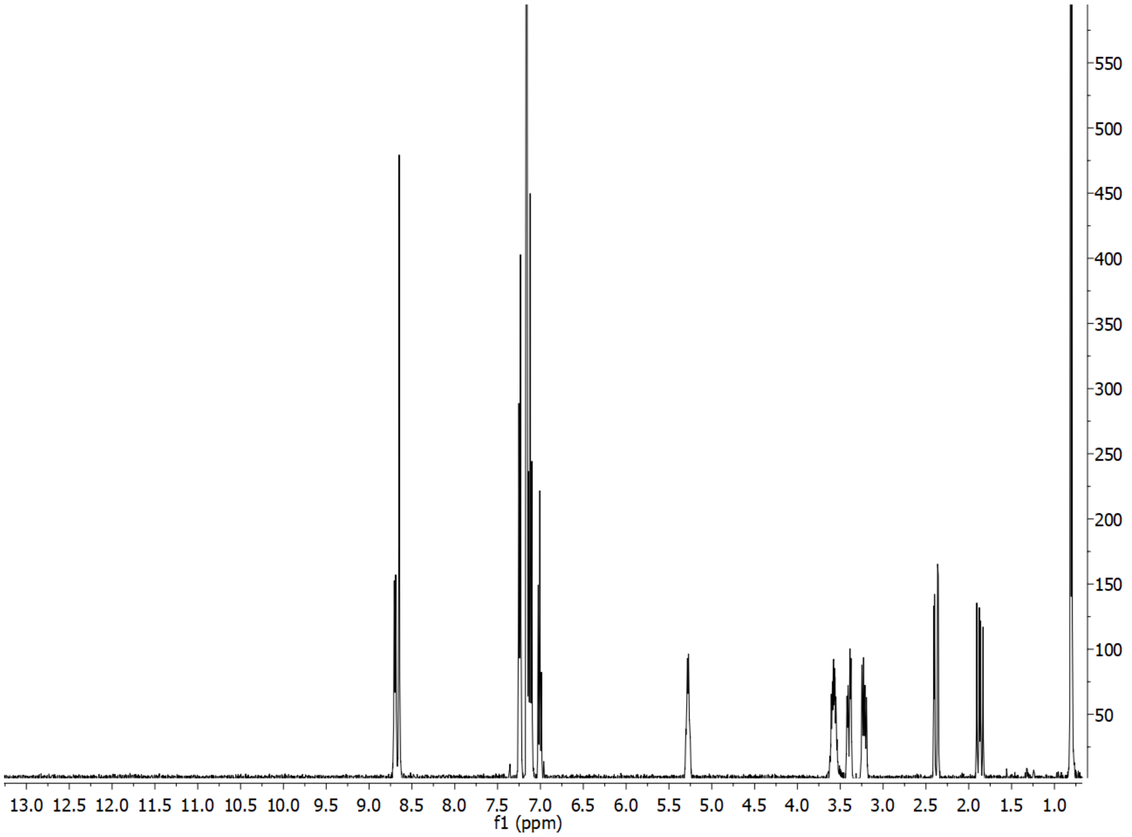

Figure S2. ^{1}H-NMR (400 MHz, Benzene-d_6) spectra of ochratoxin A. Ochratoxin A: ^{1}H NMR (400 MHz, Benzene-d_6) δ 8.70 (H-10, d, *J* = 6.8 Hz, 1H), 8.65 (H-6, s, 1H), 7.24 (H-5′+H-9′, d, *J* = 7.5 Hz, 2H), 7.12 (H-6′+H-8′, t, *J* = 7.5 Hz, 2H), 7.01 (H-7′, t, *J* = 7.4 Hz, 1H), 5.28 (H-2′, m, 1H), 3.58 (H-3, ddd, *J* = 11.9, 6.1, 3.4 Hz, 1H), 3.40 (H-3′b, dd, *J* = 14.1, 5.0 Hz, 1H), 3.22 (H-3′a, dd, *J* = 14.0, 7.3 Hz, 1H), 2.38 (H-4b, dd, *J* = 17.3, 3.4 Hz, 1H), 1.87 (H-4a, dd, *J* = 17.3, 11.9 Hz, 1H), 0.80 (H-11, d, *J* = 6.3 Hz, 3H).

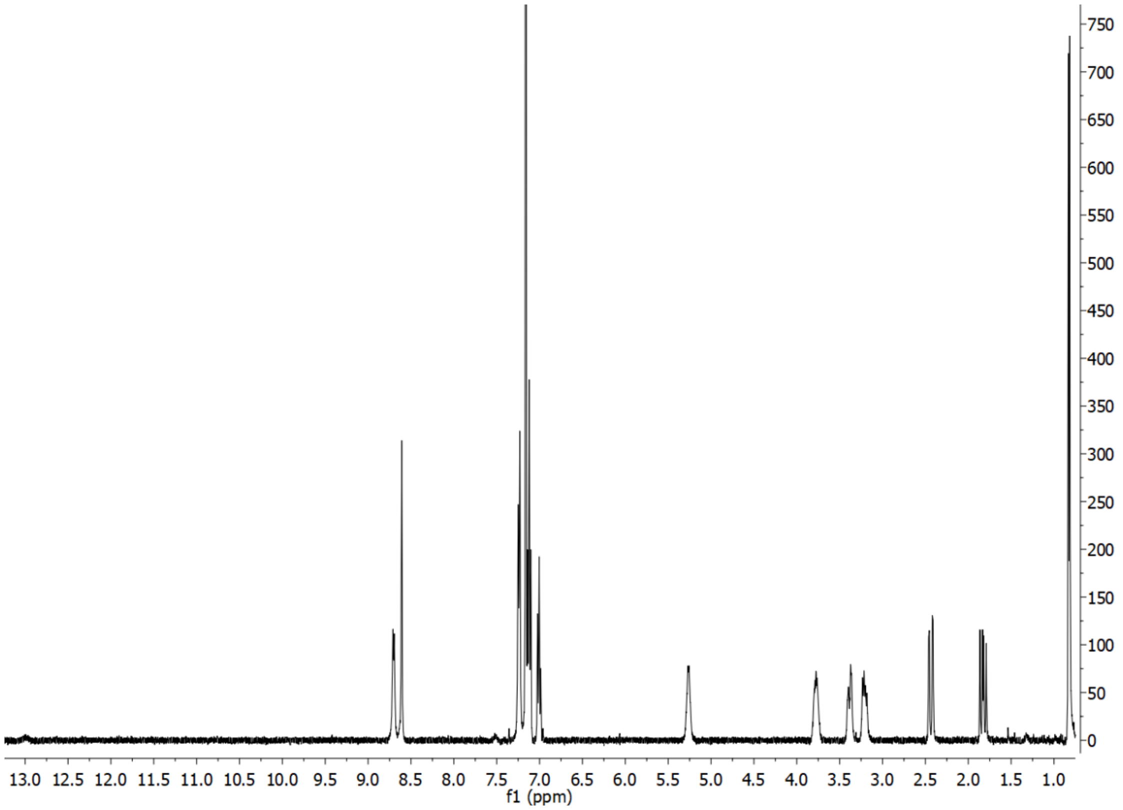

Figure S3. ^{1}H-NMR (400 MHz, Benzene-*d*$_6$) spectra of 2′*R*-ochratoxin A. 2′*R*-ochratoxin A: ^{1}H NMR (400 MHz, Benzene-*d*$_6$) δ 8.70 (H-10, d, J = 6.6 Hz, 1H), 8.61 (H-6, s, 1H), 7.24 (H-5′+H-9′, d, J = 7.5 Hz, 2H), 7.12 (H-6′+H-8′, t, J = 7.5 Hz, 2H), 7.01 (H-7′, t, J = 7.3 Hz, 1H), 5.25 (H-2′, m, 1H), 3.83 – 3.72 (H-3, m, 1H), 3.38 ((H-3′b, dd, J = 14.1, 4.6 Hz, 1H), 3.21 (H-3′a, dd, J = 14.1, 7.3 Hz, 1H), 2.44 (H-4b, dd, J = 17.3, 3.3 Hz, 1H), 1.83 (H-4a, dd, J = 17.3, 12.0 Hz, 1H), 0.82 (H-11, d, J = 6.3 Hz, 3H).

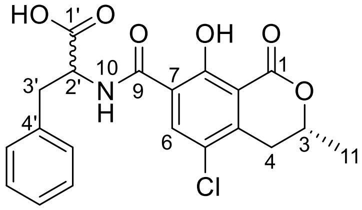

ochratoxins

Figure S4. Molecular structure of the ochratoxins OTA and 2′*R*-OTA with labeled atoms.

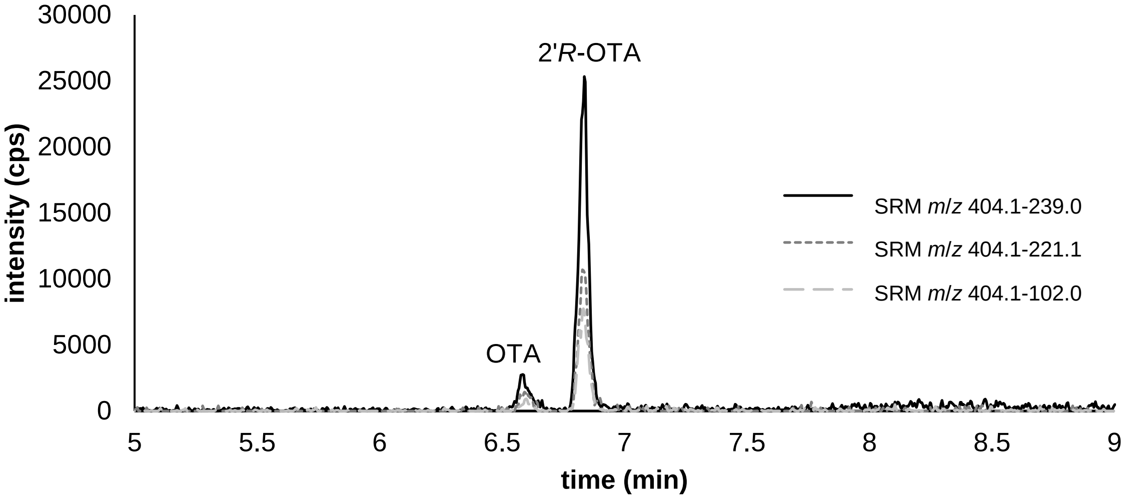

Figure S5. HPLC-MS/MS chromatogram of OTA and 2′*R*-OTA with the three transitions from the dialysis chamber without HSA after 5.5h. cps: counts per second.
